# Glycomic and glycoproteomic analysis of glycoproteins—a tutorial

**DOI:** 10.1007/s00216-017-0406-7

**Published:** 2017-06-06

**Authors:** Asif Shajahan, Christian Heiss, Mayumi Ishihara, Parastoo Azadi

**Affiliations:** Complex Carbohydrate Research Center, The University of Georgia, 315 Riverbend Road, Athens, GA 30602 USA

**Keywords:** Glycomics, Glycoproteomics, Mass spectrometry, Glycan analysis, Glycopeptide, Glycosylation site mapping

## Abstract

The structural analysis of glycoproteins is a challenging endeavor and is under steadily increasing demand, but only a very limited number of labs have the expertise required to accomplish this task. This tutorial is aimed at researchers from the fields of molecular biology and biochemistry that have discovered that glycoproteins are important in their biological research and are looking for the tools to elucidate their structure. It provides brief descriptions of the major and most common analytical techniques used in glycomics and glycoproteomics analysis, including explanations of the rationales for individual steps and references to published literature containing the experimental details necessary to carry out the analyses. *Glycomics* includes the comprehensive study of the structure and function of the glycans expressed in a given cell or organism along with identification of all the genes that encode glycoproteins and glycosyltransferases. *Glycoproteomics* which is subset of both glycomics and proteomics is the identification and characterization of proteins bearing carbohydrates as posttranslational modification. This tutorial is designed to ease entry into the glycomics and glycoproteomics field for those without prior carbohydrate analysis experience.

## Introduction

The study of glycoproteins is a rapidly growing field, which is not surprising considering that at least half of all proteins in living organisms are equipped with oligosaccharide chains (“glycans”) [1]. These glycans influence many physiological interactions, directly or indirectly impacting the functioning of cells. To understand these functions, it is necessary to know the precise glycan structure, their structural variability, their sites of attachment to the protein, and the degree to which these sites are occupied. These structural assignment tasks are often made difficult by several factors characteristic of glycoproteins, such as nontemplate-driven biosynthesis and microheterogeneity [2]. The biosynthesis of glycans is driven by a set of glycosyltransferase-based cascades, and therefore, glycans on a particular site always exist as mixtures of similar structures, often containing groups of isomers. Structural determination of carbohydrates from complex biological samples is based on analytical methodologies such as nuclear magnetic resonance (NMR), electrospray ionization-mass spectrometry (ESI-MS), matrix-assisted laser desorption ionization MS (MALDI-MS), and capillary electrophoresis (CE), developed as the most popular approaches in both academia and industry [3]. While mass spectrometry is best suited for the determination of chain length and composition of monosaccharide classes, linkage information and monosaccharide identification are usually deduced using gas chromatography (GC) (after chemical derivatization) or NMR. In order to enable high-throughput analysis of a complex glycome, these complimentary techniques are often used together, and they are often integrated with data analysis platform tailored for the rapid sequencing of biomolecules based on certain key analytical signatures [4, 5].

However, since glycoproteins are usually available as a heterogenic mixture in minute amounts, mass spectrometry has risen to the top of the techniques since it can in principle be used for the analysis of complex mixtures and for low-abundance samples. Although mammalian glycans consist of a limited assortment of monosaccharides, these are often isomeric, having exactly the same molecular mass. The glycans can be multiply branched and usually exist as mixtures of various branching and substitution patterns [6]. Because of the presence of several potential attachment points in each monosaccharide, multitudes of structures are possible. However, as mentioned above, stereoisomers, for example mannose and galactose, are not distinguishable based on their molecular mass. Although some of those stereoisomers produce slightly different ring cleavage pattern in tandem MS^*n*^ analysis, it is more common to combine other analytical techniques to distinguish the stereoisomers, such as glycosyl linkage or composition analysis by GC-MS of partially methylated alditol acetates (PMAAs) generated from glycans [7].

Glycans are usually found on the cells in the form of glycoproteins or glycolipids, where they are covalently attached to either proteins or lipids, respectively. Since the focus of this tutorial is on the structural characterization of glycans associated with proteins, only protein glycosylation is discussed. The linkage connecting two monosaccharides is called “glycosidic bond.” Glycans are attached to the proteins primarily by one of two major linkages, the one involving linkage of the glycan to the protein through the side chain nitrogen of asparagine (N-linked glycans) and the other involving linkage through the side chain oxygen of serine or threonine (O-linked glycans). In the case of N-linked glycosylation, glycosyltransferase-mediated en bloc transfer of oligosaccharide takes place between a lipid-linked oligosaccharide to the acceptor asparagine of nascent proteins containing the consensus amino acid sequon Asn-X-Thr/Ser (X is any amino acid except proline). The most common O-linked glycosylation is initiated with the addition of a single monosaccharide GalNAc (mucin-type glycosylation) or GlcNAc (O-GlcNAcylation) to serine or threonine of proteins irrespective of any defined sequence motif, and this monosaccharide is further elongated in the case of GalNAc to form various core glycan structures [6].

Since glycans play a part in almost all biological processes such as intra- and intercellular signaling, organ development, immunological responses, tumor growth, and even stability of bioconjugates, a comprehensive analysis of cellular glycan repertoire is essential for the study of underlying mechanisms in these complex biological processes. Presently, various levels of analytical characterization of glycosylation have been proposed to understand this discrete and dynamic modification of biomolecules. The first level comprises the analysis of the individual glycan structures in detail along with their isomeric pattern (glycomics). The second level involves detailed evaluation of site of glycosylation on glycoproteins and glycopeptide characterization (glycoproteomics), including the glycan variability and degree of occupancy of the site. The third level consists of studying the glycomic profile in different cellular and tissue systems and the influence of glycan structure in order to understand cellular communication [6]. Only the first two levels are discussed in this tutorial.

Matrix-assisted laser desorption ionization-time-of-flight mass spectrometry (MALDI-TOF-MS) (see “Matrix-assisted laser desorption ionization-MS” section), which is one of the most common techniques for glycan characterization, enables rapid and sensitive analyses of singly charged larger biomolecules [8]. However, MALDI-TOF-MS analysis of native glycans is challenging due to their structural complexity and low ionization efficiency, which is a result of the hydrophilicity of carbohydrates. Permethylation of glycans improves the sensitivity for mass spectrometry detection by increasing the ionization efficiency of glycans up to 20-fold [9]. Further structural characterization of selected glycan ions is possible by electrospray ionization mass spectrometry (ESI-MS) (see “Electrospray/nanospray ionization-MS” section) and tandem (MS^*n*^) mass spectrometry fragmentation techniques. This analysis allows differentiation between “isobaric” glycans that have the same mass but different sugar compositions, linkages, or structures [10]. Liquid chromatography-MS^*n*^ (LC-MS^*n*^) analysis (see “Liquid chromatography-MS” section) of permethylated glycans offers more leverage in obtaining the fragmentation information and structural determination of isomers. More recently, techniques such as isomer differentiation of permethylated glycans through LC by better chromatographic separation system and trapped ion mobility spectrometry (TIMS) was reported [10, 11].

This tutorial will provide a brief overview, as well as concise explanations of the general approaches and individual steps to determine glycan structure, sites of glycosylation, site-specific glycan heterogeneity, and glycosylation site occupancy of glycoproteins by using mass spectrometric techniques. References to published manuscripts that describe established protocols in detail will be provided for each experiment. In selecting the methods to be included here, we limited ourselves to those protocols that we ourselves routinely use in our service facility. These methods are time-tested and robust and will provide reliable data in the vast majority of cases. Anyone who is attempting glycomics or glycoproteomics analysis using the methods presented in this tutorial and is experiencing difficulties is invited to contact us, and we will do our best to guide them through the process. The tutorial is not meant to be a comprehensive review covering every method that has been developed recently, but rather as an introductory guide to successful glycomics and glycoproteomics. The goal of this tutorial is to provide a tool to enable nonspecialists to determine whether glycans are important in their research, and if they are, how to get started in obtaining a glycomic and glycoproteomic description of their samples.

## Overview

**Fig. 1:**
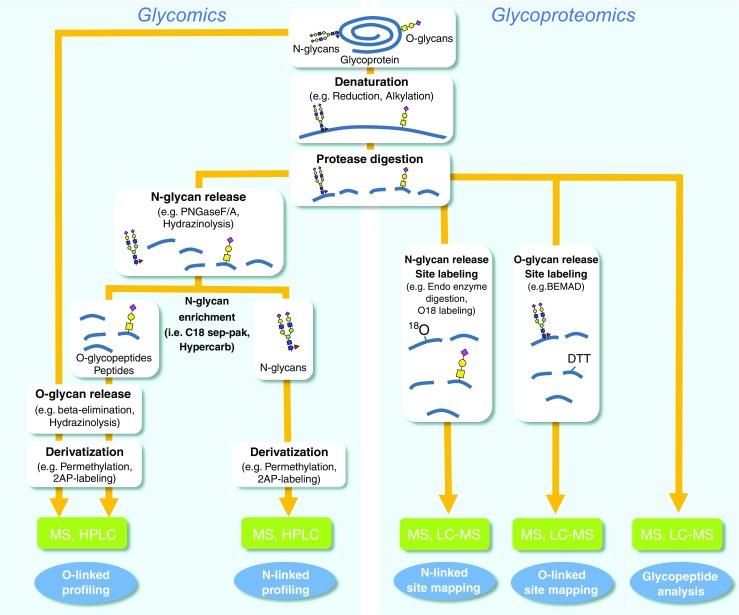
General workflows for glycomics and glycoproteomics analysis. Glycomics involves the release of N-linked glycans (using PNGase F, PNGase A, or hydrazinolysis) and O-linked glycans (by reductive alkaline β-elimination or hydrazinolysis) and subsequent derivatization of glycans (by permethylation or reductive amination with chromophores such as 2-AP) and analysis by MALDI and ESI mass spectrometry. Glycoproteomics comprises of determination of glycosylation at glycopeptide through direct LC-MS/MS analysis of intact glycopeptide and identification of site of glycosylation of O-glycans (BEMAD or ETD) and N-glycans (^18^O-labeling, endo-H digestion, or ETD) by tandem LC-MS/MS

The structural analysis of protein glycosylation is generally performed with or without release of the glycan modification from the glycoproteins: the former approach is termed “glycomics,” and the latter is termed “glycoproteomics” (Fig. 1). Releasing the glycans in the glycomics approach enables direct introduction into MS instrument and allows higher dimensional MS analysis (MS^*n*^). It also provides an opportunity to couple other derivatization and analytical techniques such as fluorescence labeling followed by HPLC profiling, NMR, permethylation followed by glycosyl linkage analysis (see “Linkage analysis” section) for in-depth structural characterization [12]. The downside of the glycomics approach is that site-specific information, i.e., the attachment site and occupancy rates, is lost once the glycan is released from the protein. Conversely, in the glycoproteomics approach, the glycans are not released, and the glycan-peptide bonds are carefully kept intact to obtain information about glycosylation sites and site occupancies. While efforts are underway to develop robust methods to perform both of these parts on the same sample, such procedures still face challenges in the determination of the exact structure of glycosylation on glycoproteins. Even though analysis of intact glycopeptides by LC-MS/MS is the most common way of rapid determination of glycosylation at specific site of peptides, this method is currently not sufficient to understand exact glycosylation. This is due to several factors such as relatively poor ionization of glycopeptide with respect to peptide, the presence of a multitude of glycan isomers (glycoforms), lack of a comprehensive structural database of glycans (including microbial and plant-derived structures), and inability to obtain mass spectrometric signature fragment ions needed for complete structure determination. Also, although there are currently a number of bioinformatics tools available for glycopeptide analysis, searching for highly heterogeneous glycan attachment on peptides is still extremely challenging [8, 13]. Consequently, these challenges make it currently necessary to split the sample into two separate workflows for the comprehensive characterization of glycan structure on glycoproteins, through glycomic and glycoproteomic analysis. Figure 1 outlines the steps that are included in these two workflows. The first step in both involves proteolytic cleavage of glycoprotein to obtain peptides and glycopeptides. This is required for glycoproteomic analysis because glycopeptides are the units that are being analyzed, but proteolysis is also beneficial for glycomic analysis as a preliminary step for the following enzymatic N-glycan release, because glycan release is more efficient from glycopeptides than from intact or denatured glycoproteins due to decreased steric hindrance [14, 15]. After this step, the sample is split into two parts: one for glycomic and one for glycoproteomic analysis. For the glycomic analysis, N-glycans are then released from the glycopeptides using an N-glycanase enzyme, which cleaves the N-linked glycans from the asparagine residues of the peptide. The released N-glycans, which are hydrophilic, can easily be separated from the O-linked glycopeptides and nonglycosylated peptides using a C_18_ solid-phase extraction (SPE) cartridge or nonporous graphitized carbon column [7]. Subsequently, the O-linked glycopeptides and nonglycosylated peptides are eluted from C_18_ SPE. Release of O-linked glycans is usually accomplished chemically because of lack of the deglycosylation enzymes with wide specificity for O-linked glycans. The common chemical procedures used for releasing O-glycan include reductive β-elimination, ammonia-based nonreductive β-elimination, or hydrazinolysis [15–17]. In order to enable highly sensitive detection, O-linked and N-linked glycan fractions are usually derivatized prior to mass spectrometric analysis, either by permethylation or by reducing-end labeling with chromophores (e.g., 2-aminobenzamide (2-AB), 2-aminopyridine (2-AP), 4-aminobenzoic acid, or anthranilic acid) [18].

Derivatization enhances the ionization of the released glycans, and permethylation, in particular, enables extraction of more detailed structural information from the MS/MS spectra through both glycosidic and cross-ring cleavages [19]. Thus, we are highlighting in this tutorial the glycan structural characterization by employing permethylation as derivatization approach, of which the procedures are essentially the same for both N- and O-glycans. The portion of the permethylated fractions that was not used for mass spectrometry can be further manipulated for linkage analysis by GC-MS, which identifies each of the glycan’s monosaccharides and their connection points. During this step, the permethylated glycans are acid hydrolyzed, reduced, and acetylated, and the resulting PMAAs are analyzed by GC-MS [7].

Linkage analysis does not provide an accurate quantification of monosaccharides, which can be accomplished by glycosyl composition analysis, where monosaccharides derived from glycans by acidic methanolysis are derivatized by trimethylsilyl (TMS) groups and analyzed by GC-MS [20]. An alternate method for monosaccharide composition analysis is high-performance anion exchange chromatography with pulsed amperometric detection (HPAEC-PAD) of monosaccharides derived from glycans by acid hydrolysis [21]. These procedures can be carried out at any stage before permethylation.

Glycoproteomic analysis consists of two complimentary workflows: glycosylation site mapping and glycopeptide analysis. Site mapping is usually performed first because it reveals the potential glycosylation sites that are occupied. This information is very useful for data analysis in the subsequent glycopeptide analysis. Since several analytical challenges are associated with deducing the glycosylation site from intact glycopeptides, notably lack of peptide fragmentation information during MS/MS, many researchers prefer to perform analysis on deglycosylated peptides or partially deglycosylated peptides. Some of these techniques involve the enzymatic removal of N-linked glycans with peptide-N-glycosidase (PNGase) in ^18^O-labeled water or partial enzymatic degradation of the N-linked structures facilitated by endo-β-N-acetylglucosaminidase (see “N-linked site mapping” section) [22]. On the other hand, in most cases, the sites of O-linked glycans can be determined without release because O-glycans are smaller in size than N-glycans in general, and therefore, allow a greater chance to obtain peptide fragmentation, which are essential for site determination by tandem MS analysis. However, if the glycoprotein carries large O-glycans or is heavily glycosylated (i.e., mucin), removal of O-linked glycans may be necessary for site mapping. A site mapping method termed BEMAD (β-elimination by Michael addition with dithiothreitol), a mildly alkaline β-elimination in the presence of dithiothreitol (DTT), is one of the possible approaches to accomplish release of O-glycans with simultaneous site labeling (see “O-linked site mapping by BEMAD” section) [23]. The glycopeptides or deglycosylated and labeled peptides can be analyzed directly by mass spectrometry (i.e., MALDI-TOF-MS or ESI-MS) or they can be separated first by LC in LC-MS. In LC-MS analysis, the peptides, glycopeptides, and labeled peptides are first separated on LC and then injected on-line into the high-resolution mass spectrometer for the mass measurement of each component and its fragments. Although it is possible to gain information about both the glycans and their attachment sites from glycoproteomics data, a more complete characterization requires the use of glycomics results. The information from the glycomic analysis and the site mapping experiment can also be quite helpful for glycoproteomic data analysis, narrowing the range of possible masses to look for in the glycopeptide analysis [19]. The final important aspect in both glycomics and glycoproteomics analysis is the data interpretation of multiple types of tandem MS^*n*^ data. This is achieved through various platforms of available bioinformatics tools comprising several databases curated through experimental data, in silico fragmentation prediction tool, search algorithms, annotation tools, and glycan structure drawing tools [24–28].

## Step-by-step description of workflows

### Protein/glycoprotein extraction

A glycomics study can be accomplished from a single purified glycoprotein, glycoprotein mixture, gel slices, and also extracts from cells, tissues, and culture supernatants. When starting the glycomics study from cells or tissues, the material should be homogenized first. There are several ways to homogenize the material, for example, dounce homogenization, enzymatic digestion, use of detergent, use of liquid nitrogen, or ultrasonication. After homogenization, proteins/glycoproteins in the homogenate can be enriched by protein precipitation by adding an organic solvent such as a mixture of chloroform and methanol, or ethanol, or acetone to the homogenate. The protein-rich precipitate formed is recovered by centrifugation and can be used for mass spectrometric studies [29].

### Protease digestion

Most glycoprotein analysis follows the bottom-up approach, i.e., the glycoprotein is cleaved into smaller units, rather than the top-down approach that analyzes the whole glycoprotein. Although advances in instrumentation have allowed progress in top-down glycoproteomics, this tutorial is limited to the far more common bottom-up approach. In this context, both glycomics and glycoproteomics analysis involves cleavage of the glycoprotein into smaller peptides by protease(s) at an early stage in the workflow (Fig. 1) [7, 15, 30, 31]. In order to favor complete protease digestion, disulfide bridges in the protein are broken by reduction with DTT, tris(2-carboxyethyl)phosphine (TCEP), or 2-mercaptoethanol. The reduction is usually followed by alkylation with iodoacetamide (“carbamidomethylation”) or iodoacetic acid (“carboxymethylation”) to prevent reformation of disulfide bond. The protease digestion is usually done by a protease enzyme or combination of enzymes, such as trypsin, Glu-C, chymotrypsin, etc., which should be selected based on the protein sequence of the target protein if the sequence is available [32]. In the case of glycopeptide analysis, it is necessary to select the enzyme that produces target peptides within the optimum range in size, polarity, and charge state for mass spectrometry detection [32, 33].

Protease digestion can be carried out in-solution or in-gel [7, 34, 35]. For in-gel digestion, the gels are cut into small pieces (∼1 mm^3^), destained by washing the gels through repeatedly swelling with ammonium bicarbonate buffer/acetonitrile (for Coomassie-stained gels), followed by reduction and alkylation of the protein in gel. After washing out the chemicals introduced, the protein in gel is digested with protease(s). The resulting peptides and glycopeptides can be extracted with increasing proportions of acetonitrile acidified with formic acid. Before going to the next step, it is important to deactivate the protease after protease digestion to prevent it from degrading the N-glycanase. For in-solution digestion, proteins are reduced, alkylated, and subsequently desalted by dialysis, ultrafiltration, or SPE. The proteins are further digested by protease(s) in a suitable digestion buffer. The protease(s) is inactivated after digestion by pH shift, ultrafiltration, or heating [7, 36].

### Glycomics analysis

#### N-glycanase digestion

**Fig. 2:**
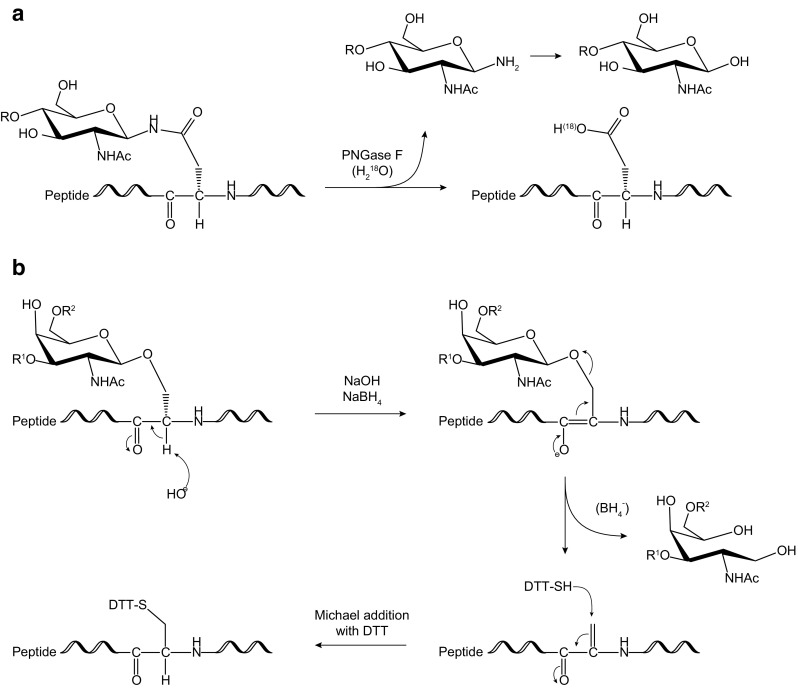
Schematic representation showing mechanism of glycan release from proteins of **A** N-glycans by PNGase F and optional ^18^O-labeling [22] and of **B** O-glycans by reductive β-elimination and optional BEMAD [23]

The N-linked glycan on the majority of glycoproteins/glycopeptides can be released with PNGase F purified from *Flavobacterium meningosepticum*, which cleaves the bond between the first GlcNAc residue of the glycan and the glycosylated asparagine (Fig. 2A) [14, 32, 34]. PNGase F does not release N-glycans bearing a core α-1-3-fucose efficiently and is also ineffective for many microbial N-glycans [37]. A similar enzyme, PNGase A from almonds, can release core α-1-3-fucosylated N-glycans, but the cleavage efficiency of PNGase A is not as high as PNGase F. Thus, PNGase F is usually the first choice if the source of the glycoprotein is mammalian. On the other hand, PNGase A is recommended if the protein is from plant or insect [7]. For the release of glycans from bacteria or any organisms expected to have unique glycosylation, a chemical procedure such as hydrazinolysis is used instead of enzymatic procedures [38].

#### Solid-phase extraction

The N-glycanase digestion results in a mixture of released N-glycans, peptides, and O-linked glycopeptides. This mixture can be separated into two fractions: one containing only the released N-glycans and one containing peptides and O-linked glycopeptides. This separation is needed to obtain clean spectra and leads to increased sensitivity, a critical parameter for low-abundance glycans. It is easily accomplished with SPE using a C_18_ cartridge, or nonporous graphitized carbon column [7]. SPE is performed with 5% acetic acid as solvent and initial extractant. The hydrophilic N-glycans are not retained on the cartridge and are collected in the flow-through fractions. The more lipophilic peptides and O-linked glycopeptides are retained and are eluted with increasing proportions of 2-propanol in 5% acetic acid [34]. Separation can also be done using a nonporous graphitized carbon column, which efficiently removes salts, SDS, and N-deglycosylated peptides, but released N-glycans may coelute with O-linked glycopeptides [7]. Thus, graphitized carbon is complementary to C_18_, and sometimes, it may be necessary to use both, e.g., for samples with high salt content.

#### Reductive β-elimination

While most N-linked glycans can be released enzymatically, commercially available enzymes for the release of O-glycans have narrow site specificity. Therefore, O-linked glycan release is usually accomplished chemically, most commonly by reductive β-elimination (Fig. 2B). The cleavage is base catalyzed using sodium hydroxide (NaOH) [7, 35, 39]. However, the newly created reducing end of the released glycan is sensitive to base, which can cause degradation through the “peeling reaction,” a repetitive β-elimination type reaction that can deconstruct the oligosaccharide from its reducing end. This degradation is prevented by adding a reducing agent to the mixture to convert the residue at the reducing end of the glycan into an alditol, which is stable to base [19, 39].

#### Permethylation

**Fig. 3:**
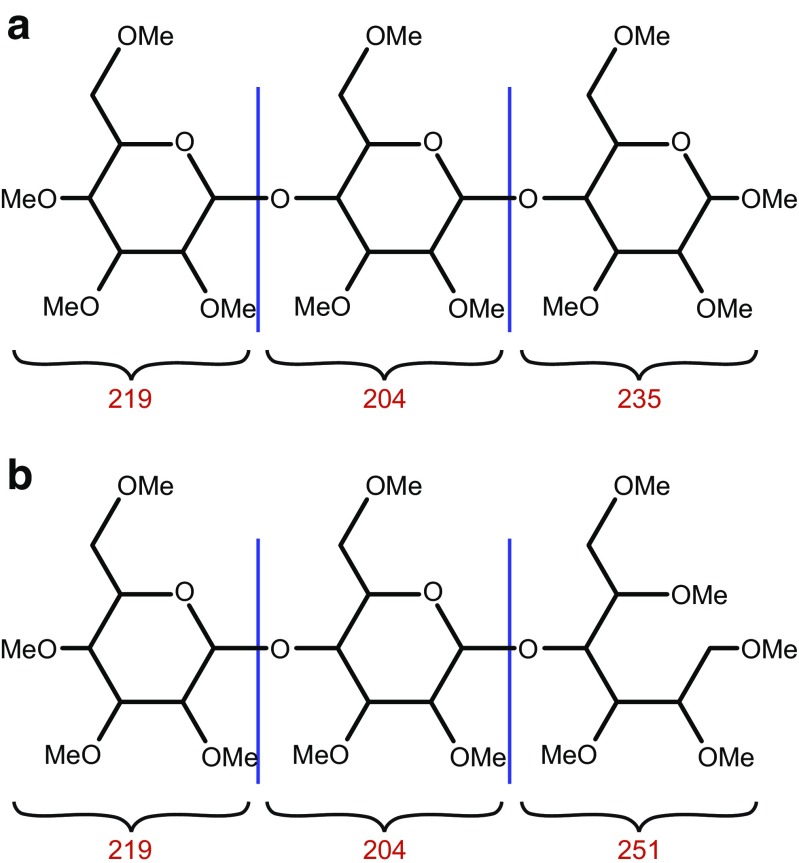
Schematic representation showing the masses of each monosaccharide component on a permethylated glycan. **A** Nonreduced permethylated glycan. **B** Reduced permethylated glycan

**Fig. 4:**
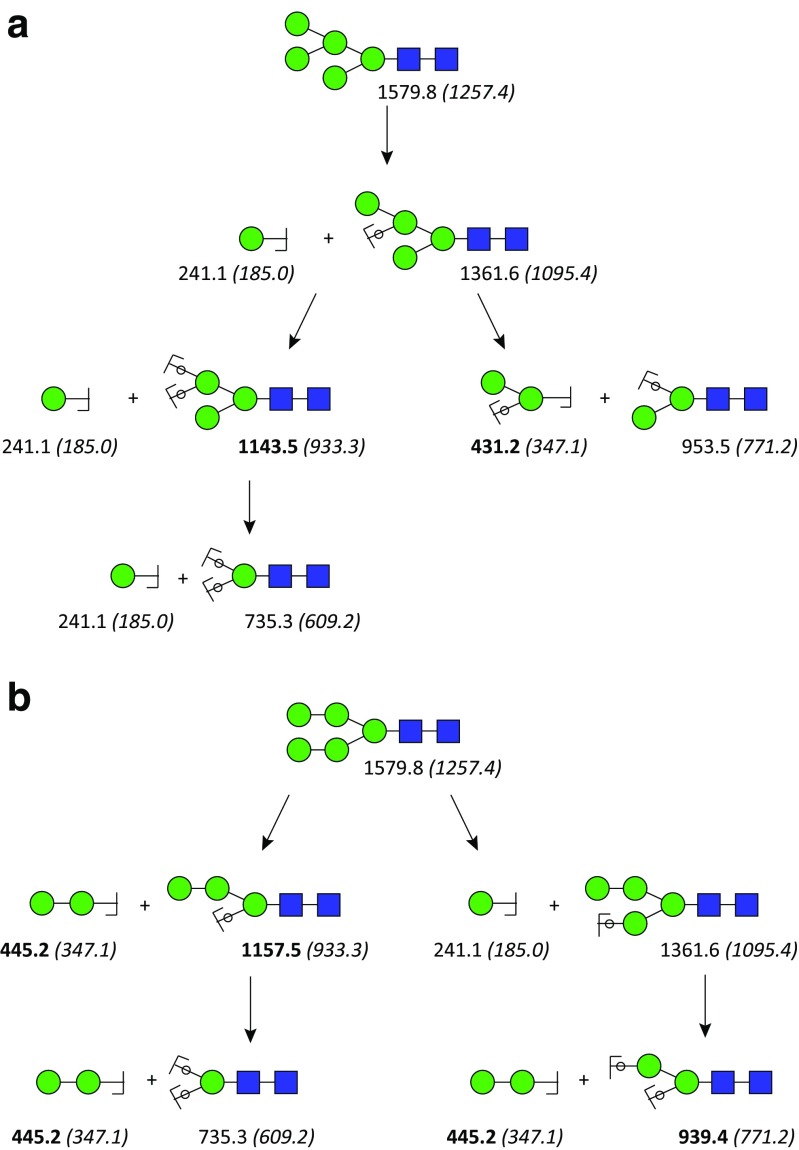
Advantage of permethylation of glycan in the differentiation of glycan isomers via CID MS^*n*^. The *m*/*z* value of sodium adduct of permethylated glycans and native glycans of N-glycan Man_5_GlcNAc_2_ (in *brackets*) which are provided under each oligosaccharide fragments shows that only MS^*n*^ of permethylated glycans possess key fragments (in *bold*) that can help to differentiate isomers **A** and **B**. The *circles* superimposing the bonds mean that the oxygen atoms of these glycosidic bonds are included in the fragment, i.e., they are Y-ions (see Fig. 9B) [41]

Permethylation (replacing every hydroxyl proton with a methyl group) is one of the most common derivatization techniques for the mass spectrometric characterization of released N- and O-glycans **(**Fig. 3) [19, 40]. Permethylation offers several advantages such as (i) reducing the hydrophilicity of glycans, which leads to reduced ion suppression, making the analysis more sensitive (10 to 20 times over native glycans); (ii) converting negatively charged carboxylate residues on glycans to neutral methyl esters, enabling the complete glycomic profile analysis in positive-ion mode; (iii) enabling comprehensive structural characterization of glycans by enhancing cross-ring fragmentation during tandem MS procedures; and (iv) tandem mass spectrometry of permethylated glycans, unlike that of native glycans, also provides information about the sequence of monosaccharides and through which positions they are linked to each other in the glycan. This is because linked positions of oligosaccharides do not become methylated, creating a 14-mass unit (CH_2_) deficit (“scar”) that can be localized by MS/MS (Fig. 4). Sensitivity enhancement can also be accomplished by reducing-end labeling the glycans with fluorescent tags, such as 2-AB, 4-aminobenzoic acid, or anthranilic acid, but these labels do not provide the same wealth of structural information as permethylation does [18, 19].

The most widely used methylating agent is iodomethane, which is employed under basic conditions. The choices of base are NaOH powder [42], DMSO-NaOH pellet [40], or methylsulfinyl carbanion [43]. The permethylation is carried out in DMSO solution, by first deprotonating all the hydroxide groups with the base and then methylating with iodomethane. After this reaction, which is usually complete within 10 min, water is added, and the permethylated glycans are desalted and extracted by water/dichloromethane phase separation or C_18_ SPE [7, 19, 34]. SPE separation is especially important for sulfated glycans [44] because the sulfates are not methylated and remain negatively charged and thus cannot be extracted with dichloromethane. In contrast, phosphate groups are mono- or di-methylated, allowing their differentiation from sulfate groups [45], which is difficult on native phosphorylated or sulfated glycans since sulfate and phosphate have nearly identical monoisotopic mass.

#### Linkage analysis

**Fig. 5:**
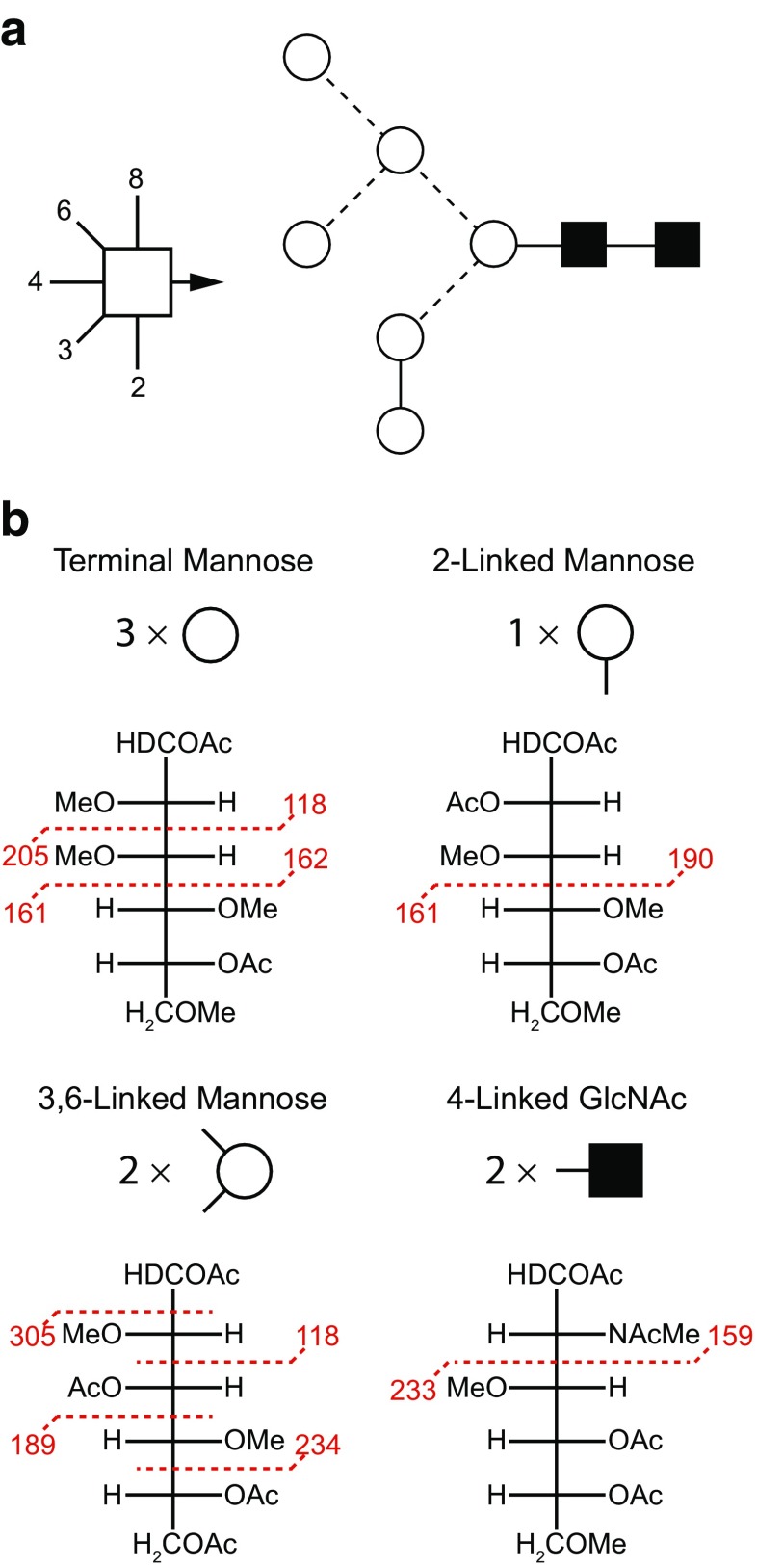
Determination of linkage positions of glycans through permethylation: permethylated glycans are acid hydrolyzed, acetylated, and reduced, and the resulting PMAAs are analyzed by GC-MS. In the PMAAs, the location of acetyl groups indicate linkage positions, whereas methyl groups indicate positions that were unsubstituted in the original glycan. During MS, fragmentation occurs preferentially next to methyl groups. The example here is a Man_6_GlcNAc_2_ N-glycan. The symbolism shown in **A** is explained in Fig. 10. The MS fragmentations are shown in **B**

In addition to analyzing the permethylated glycans by MS, they can also be further derivatized for linkage analysis (Fig. 5) [7, 48]. The permethylated glycans are hydrolyzed with acid, which cleaves them into their constituent monosaccharides, and the previously linked oxygens are converted to hydroxyls, whereas the oxygens that were not involved in linkages remain methylated. After reduction of all the monosaccharides into alditols, the free hydroxyls are O-acetylated, generating PMAAs, with methylation and acetylation patterns that are characteristic for each type of monosaccharide and each linkage position. In GC-MS analysis, the PMAAs fragment preferentially between carbons bearing O-methyl groups, which gives rise to fragmentation spectra that specify the position of the O-acetyl group and hence of the linkage positions. PMAAs originating from different monosaccharides with the same linkage pattern are distinguished by their characteristic retention times in GC [7].

#### Exoglycosidase digestions

The linkages present in a glycan can also be determined by sequential removal of nonreducing end residues by means of digestion with linkage-specific exoglycosidases. Practically, released glycans are treated with exoglycosidases sequentially. After each digestion, a small portion of the reaction mixture is taken, cleaned by SPE using a C_18_ cartridge, permethylated, and examined by mass spectrometry to observe the changes in glycan composition. Together with the knowledge of each enzyme’s specificity, this information is used to determine linkages and monosaccharide sequence [7, 49, 50].

### Glycoproteomics analysis

#### N-linked site mapping

There are two common ways to map the site of N-glycosylation. One is using endo-F or H enzyme, which releases N-glycans but leaves a GlcNAc core attached to the glycopeptide [19, 51]. By mapping the location of the remaining GlcNAc by LC-MS, we can gain information of the site location as well as the site occupancy. Due to the limited site specificity of the endo-F or H enzymes, not all N-glycans are cleaved, making this approach not generally applicable [52] [53].

By far, the most widely accepted way to map the site of N-glycosylation is ^18^O-labeling (Fig. 2A). Enzymatic deglycosylation converts asparagine residues at the N-glycosylation site of the glycopeptide to aspartic acids via a deamidation mechanism by the addition of an oxygen atom from the surrounding water. This increases the mass of the peptide by 0.9840 Da on the N-glycosylation site and thus enables identification of sites of glycosylation. However, added confidence may be attained by performing this deglycosylation reaction in “heavy” water (^18^O water), causing a mass increase of 2.9882 Da. The location of the ^18^O-labeled aspartic acid residues is then mapped by LC-MS [22]. For successful ^18^O-labeling to map the N-linked sites, it is critical to ensure that the mass shift is introduced only by PNGase F and to minimize chemical deamidation. The latter is known to be accelerated at high pH and high temperature, under which conditions asparagine can be converted into aspartic acid and *iso*-aspartic acid. To suppress such chemical deamidation during ^18^O-labeling, the experiment should be performed under slightly acidic conditions (pH 6.8) even though the optimal pH of PNGase F is slightly basic. Another pitfall of ^18^O-labeling is carry-over protease activity during the experiment, which can result in a 5- or 7-Da instead of a 3-Da mass shift of the labeled peptide, because the carry-over protease activity introduces ^18^O into the carboxyl group at the C-terminus. To prevent this, the sample should be treated with regular water or protease with regular buffer again after ^18^O-PNGase F digestion and before LC-MS analysis [19, 36].

#### O-linked site mapping by BEMAD

The reductive β-elimination of O-glycans leaves behind a modified serine or threonine residue in the former glycopeptide. This residue is reactive toward the thiol functionality, and this property is exploited in the BEMAD method in order to tag this residue for unambiguous MS detection. The reagent of choice for this purpose is DTT, which introduces a 136.0017-Da mass shift compared to non-O-glycosylated serine or threonine (Fig. 2B) [23].

#### Glycopeptide analysis

**Fig. 6:**
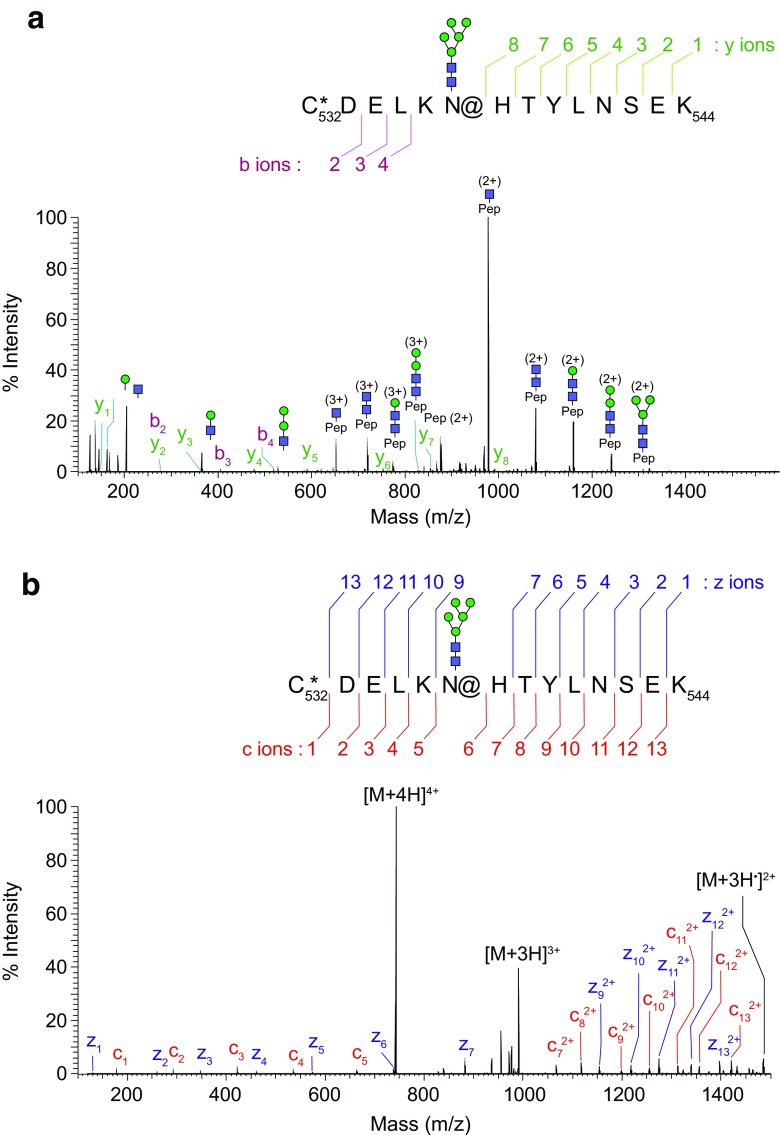
**A** An example MS/MS-HCD spectrum of a glycopeptide carrying a high-mannose type N-glycan, Man_5_GlcNAc_2_ on asparagine. It showed a series of glycan oxonium ions in the low mass region at *m*/*z* 163.0603, 204.0868, 366.1398, and 528.1929, respectively. A series of fragment ions due to neutral loss of the glycan moiety were observed as the main fragment ions in the HCD spectrum. Trace amounts of y-type and b-type peptide fragment ions were detected, confirming the sequence of the peptide backbone. **B** An example of MS/MS-ETD spectra from a glycopeptide carrying Man_5_GlcNAc_2_, showing fragment ions with minimal neutral loss of glycan moiety. All expected peptide c-type and z-type fragment ions were detected except c8 fragment ion, confirming the peptide sequence in high confidence as well as the site and mass of the glycosylation modification. An *asterisk* (*) represents carbamidomethylation of cysteine; *at symbol* (@) represents the site of N-glycosylation, respectively [55]

The mixture of N- and O-linked glycopeptides and peptides obtained in the protease digestion can be analyzed directly by LC-MS [54, 55]. Glycopeptides are characterized by tandem mass spectrometry, often referred to as “MS/MS” or “MS^*n*^,” through which fragmentation by appropriate dissociation techniques is performed to obtain several key fragments characteristic of the overall glycopeptide structure (Fig. 6). Thorough elucidation of glycopeptide structure requires simultaneous determination of its amino acid sequence and exhaustive characterization of its carbohydrate(s), including the site of attachment (Fig. 6B) and degree of site occupancy. Due to microheterogeneity, where a glycoprotein may carry a variety of glycan structures on a given glycosylation site, several tandem MS methods such as collision-induced dissociation (CID), higher-energy collisional dissociation (HCD), or electron transfer dissociation (ETD) (see “MS fragmentation techniques” section) either separately or in combination are typically necessary for a comprehensive glycopeptide characterization [56]. In order to detect glycopeptides in the presence of many nonglycosylated peptides, the mass spectrometer software can be programmed to detect glycan-specific fragmentation such as neutral loss of glycan mass and oxonium ions of glycans (protonated carbohydrate fragments produced from the glycans in the collision cell during fragmentation). A drawback of LC-MS of peptide-glycopeptide mixtures is the much lower ionization efficiency of glycopeptides, resulting in missing a significant number of glycopeptides and hence glycosylation sites. In order to alleviate this problem, glycopeptides can be enriched by lectin affinity chromatography, hydrophilic interaction chromatography (HILIC), hydrazide capture, or TiO_2_ (sialoglycopeptides) [19, 57–60].

### Glycosyl composition analysis

Monosaccharide composition analysis can be carried out at any point in the workflow up to the permethylation step. The individual monosaccharides can be separated by various methods, including HPAEC-PAD, GC, or CE.

#### High-performance anion exchange chromatography with pulsed amperometric detection

At high pH, the hydroxyl groups of sugars become ionized, and the resulting negative charge can be exploited for separation on an anion exchange resin [21]. Neutral and amino sugars are eluted isocratically with 16 mM NaOH, and acidic sugars, such as uronic and sialic acids, are eluted with a gradient of 70–300 mM acetate in 100 mM NaOH. The eluting monosaccharides are detected with electrochemical PAD. The disadvantages of HPAEC-PAD are low resolution and the absence of mass spectra to identify the analytes. These drawbacks are usually not prohibitive in the analysis of mammalian glycoproteins because they encompass a comparatively small variety of monosaccharides. In fact, HPAEC-PAD is the preferred method for composition analysis of mammalian glycoproteins because it is sensitive, provides accurate quantification, and does not require a complicated derivatization scheme.

#### Gas chromatography-mass spectrometry

In order to make monosaccharides volatile enough to enter the gas phase at practical temperatures and without decomposition, they require derivatization of all their hydroxyl groups with nonpolar substituents. The most versatile such nonpolar substituent is the TMS group, even though several other silyl-based derivatization agents have also been developed. It can be used to volatilize neutral, acidic, and amino sugars. Most often, this derivatization scheme is started by acidic methanolysis, which produces methyl glycosides, whose remaining hydroxyl protons are then replaced by TMS groups [20]. The monosaccharides are not reduced in this method, preserving their mutarotation equilibrium that includes α- and β-anomers of both furanose and pyranose forms. As a result, each monosaccharide is detected as a mixture of at least four peaks (often more due to incomplete derivatization). This can be an advantage because it largely precludes misidentification of monosaccharides. On the other hand, the alditol acetates (AA) [61] and peracetylated aldononitrile (PAAN) [62] methods produce chromatograms with only one peak per monosaccharide. This is accomplished by reduction in the case of AA and by converting the sugar aldehyde into a nitrile in the case of the PAAN method. The reduction in the AA method converts the aldehyde into a primary alcohol that can often not be distinguished from the primary alcohol already present in many monosaccharides (e.g., primary alcohol at C-6 in glucose). This can lead to conversion of two different monosaccharides to the same alditol acetate. A widely employed solution to this problem is to use a reducing agent that transfers deuterium instead of hydrogen and thus introduces a mass label into the AA. A disadvantage of both the AA and the PAAN methods is that neither of them can be used to detect uronic acids directly [20].

### Mass spectrometry

The advent of soft ionization techniques has made it possible to obtain mass spectra of intact macromolecules. Two soft ionization techniques, in particular, have risen to the top in the analysis of carbohydrates and proteins. These are MALDI and ESI. A newer development in ESI is the usage of extremely low flow rates (on the order of nL/min) liquid chromatographic system, in which case the method is termed nanospray ionization (NSI). NSI has been shown to be more sensitive and salt-tolerant than ESI and thus facilitates the sensitive, rapid, and detailed structural analysis by obviating tedious derivatization procedures such as reductive amination or permethylation [63]. MALDI is usually combined with TOF mass analyzers, whereas ESI and NSI are frequently combined with ion-trap, Orbitrap, quadrupole, or ion cyclotron resonance (ICR) mass analyzers [19].

#### Matrix-assisted laser desorption ionization-MS

**Fig. 7:**
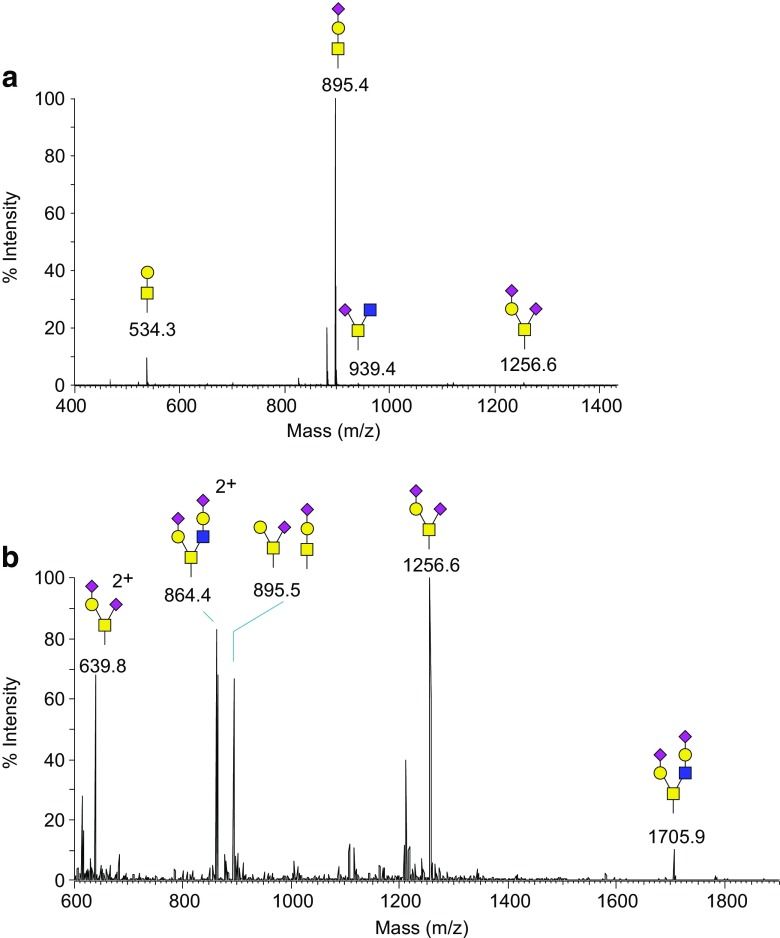
**A** MALDI-MS of released and permethylated O-glycans released from IgA. **B** Nanospray-full MS spectrum of released and permethylated O-glycans from human *siat7e*-modified MDCK cells. O-glycans were released by reductive β-elimination

For MALDI-TOF-MS, the sample solution is mixed with a “matrix” solution, and a small portion of the mixture is transferred to a stainless steel sample plate, allowed to dry, and analyzed [64]. The matrix, which is the key to a successful MALDI, is usually an organic acid (Table 1) that co-crystallizes with the sample and helps it to ionize when irradiated by intense laser pulses. Laser irradiation creates a plume containing both matrix and analyte molecules and ions. The analyte ions are mostly produced by protonation, sodiation (both in positive-ion mode), or deprotonation (in negative-ion mode) and are analyzed most commonly by TOF, quadrupole, or ion-trap mass analyzers. The method requires very little material (if permethylated, nanogram scale of oligosaccharides is detectable) and allows high-throughput analysis. Minimal sample workup is required for MALDI as the technique is relatively tolerant of salts and other nonsurfactant additives or contaminants [70, 71]. However, only one degree of tandem MS is usually possible in MALDI-TOF-MS configuration, limiting the amount of structural information that can be obtained on the glycan/glycopeptide. Another limitation to the use of MALDI for native and reductively aminated glycans is that due to relatively high degree of vibrational excitation of the ions during the ionization process, a significant degree of fragmentation occurs to fragile glycan substituents such as sialic acid and fucose residues and sulfate and phosphate groups. Nevertheless, permethylated glycans (Fig. 7A), which are ionized typically as sodium cationized ions, are relatively more stable during MALDI process than native and reductively aminated glycans [72, 73].

**Table 1:** Common MALDI matrices used in glycomics and glycoproteomics

Chemical name	Name	Solvent^a^	Notes
2,5-Dihydroxybenzoic acid	DHB	50:50 water/acetonitrile	Glycans, glycopeptides [65]
2,4,6-Trihydroxyacetophenon	THAP	50:50 water/acetonitrile with 0.1% TFA	Glycoproteins, glycopeptides [66]
α-Cyano-4-hydroxycinnamic acid	CHCA	50:50 water/acetonitrile with 0.1% TFA	Glycopeptides [65]
3,5-Dimethoxy-4-hydroxycinnamic acid	Sinapinic acid	70:30 water/acetonitrile with 0.1% TFA	Large proteins [67]
3-Aminoquinoline/α-cyano-4-hydroxycinnamic acid	3-AQ/CHCA	9:9:2 water/acetonitrile with NH_4_H_2_PO_4_	Glycans [68]
2,5-Dihydroxybenzoic acid/N,N-dimethylaniline	DHB/DMA (3:1)	1:1 water/acetonitrile	Glycans [69]

^a^Dissolve 10–20 mg in 1 mL of solvent; some matrices may not dissolve completely

#### Electrospray/nanospray ionization-MS

**Fig. 8:**
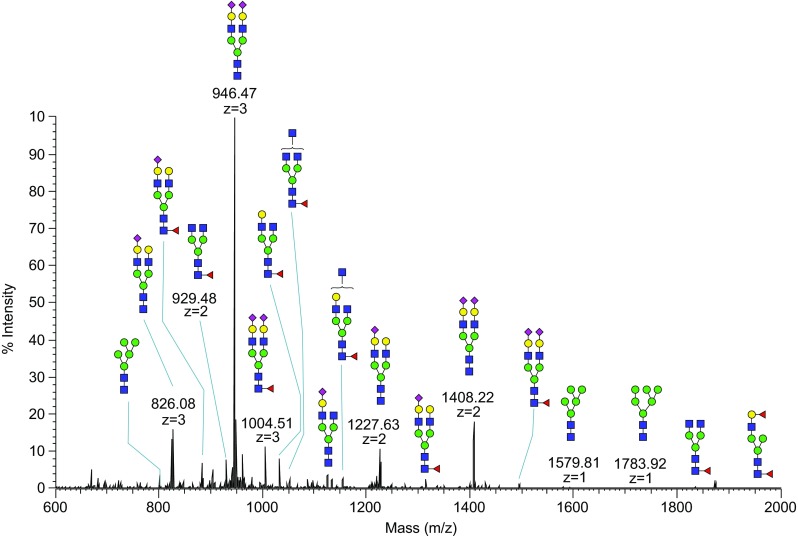
Nanospray-FT full MS spectrum of permethylated N-glycans released from human serum. N-linked glycans were released enzymatically by PNGase F, permethylated and analyzed by a direct infusion into the mass spectrometer

In ESI and NSI, a solution of the analyte exits the end of a capillary within a strong electric field. The solution forms small, charged droplets that are propelled by the field toward an ion transfer tube. On that trajectory, the droplets decrease in size through solvent evaporation, and their charge density increases until they become unstable and break apart. This process repeats until eventually individual analyte ions are produced, which enter into the increasing vacuum of the mass spectrometer through the ion transfer tube. In ESI, the solvent evaporation is usually assisted by the flow of an inert gas (“sheath gas”). The ions thus stripped of solvent molecules can then be analyzed in ion-trap, quadrupole, ICR, TOF, or Orbitrap analyzers (Figs. 7B and 8). The solvents are usually a mixture of water and methanol or acetonitrile [74]. Additives, such as formic acid for positive-ion mode and sodium hydroxide for negative-ion mode can improve the ionization efficiency in ESI/NSI. As a result of the ionization mechanism in ESI/NSI, multiply charged ions are often observed, and this allows analysis of ions that are much heavier than the detectable mass-to-charge range of the instrument. Combining ESI/NSI with ion-traps also makes it possible to do multiple levels of tandem mass spectrometry (MS^*n*^), by trapping fragments and subjecting them to further fragmentation [75].

##### Direct infusion

When the sample solution is introduced directly (via syringe pump) to the mass spectrometer without prior column separation, the method is said to be run by “direct infusion” [34]. In direct infusion, the sample composition does not change over time, and signal scans can be accumulated as long as the sample is available, increasing sensitivity. This usually also allows enough time to obtain as many levels of tandem mass spectrometry as desired. Direct infusion is the preferred method for the analysis of permethylated released glycans, as the glycan mixtures are usually well resolved in the MS. However, in order to separate isomeric glycans, LC-MS is required [19].

##### Liquid chromatography-MS

LC-MS is used for glycopeptide analysis and to separate isomeric released glycan structures [76, 77]. In order to increase sensitivity, columns with very small internal diameters are preferred. Packed capillaries can also be combined directly with the spray capillary in a single piece to reduce dead volume in NSI. The most common column packing is C_18_, but C_8_, C_4_, HILIC, and other solid phases can also be used. Due to the often small peak width in LC-MS, the time to acquire spectra is limited. Normally, a dependent scan program is designed to collect tandem mass spectra on a specified number of the most intense peaks in the spectra of each component or acquire MS/MS of maximum numbers of intense ions possible within a user-defined time period (“data-dependent scans”). Also, if the molecular mass of the target ions is known, MS/MS can be set to target specific expected masses, provided by the user in a mass list (“data-independent scans”). In order to limit the amount of redundant peptide fragmentation spectra, glycopeptides can be selected for specific fragmentation events by the presence of oxonium ions in the preceding fragmentation of the same glycopeptide [19, 63, 77].

#### MS fragmentation techniques

##### Fragment ion nomenclature

**Fig. 9:**
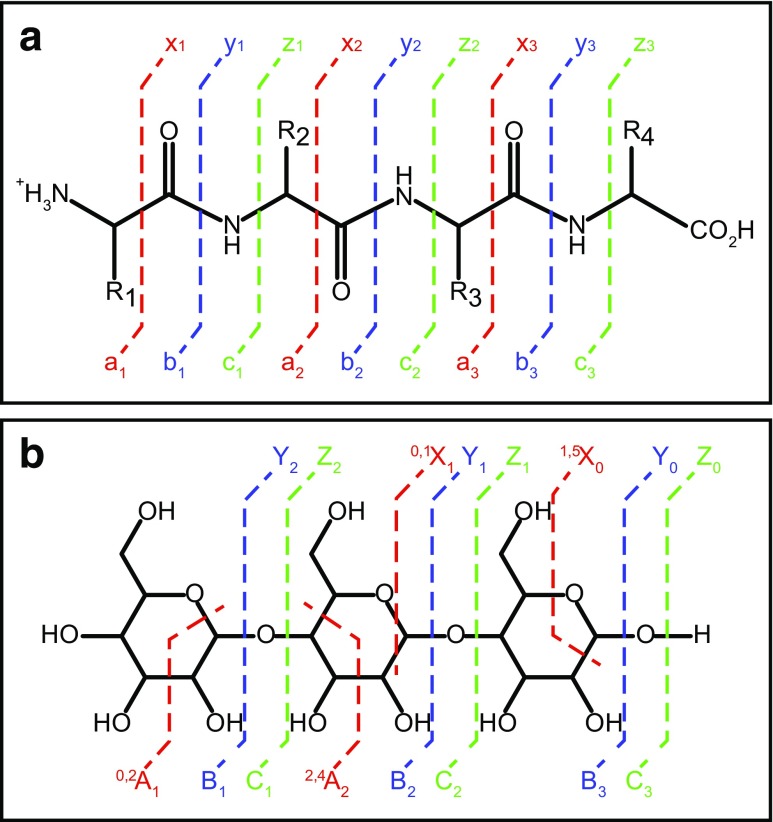
Typical fragmentation pattern and nomenclature for **A** peptides and **B** glycans, during tandem mass spectrometry

There are different types of fragmentation methods, and each one of them preferentially cleaves specific types of bonds within peptides and glycans. Nomenclature conventions have been developed to designate these fragmentation sites and resulting ions. The nomenclature for peptide fragmentations [78] is summarized in Fig. 9A, and that of glycan [79] is shown in Fig. 9B.

##### Collision-induced dissociation

CID is the oldest and still most common MS fragmentation technique. Here, the ions are activated by acceleration and then allowed to collide with a collision gas, such as helium, nitrogen, or argon. CID is an ergodic process, which means that the energy supplied by the collision is distributed within the whole ion and leads to breakage of its weakest bond. In the case of glycopeptides, this is usually a bond within the glycan moiety, and not a peptide bond, so that the predominant losses are those of the glycan chain. As a result, CID is not well suited to determine the location of glycosylation sites [80]. The fragmentation of both peptides and glycans by CID produces mostly b/B and y/Y ions. CID of permethylated glycans produces glycosidic cleavages, which allow monosaccharide composition and some sequence determination. CID fragmentation also causes cleavages of two bonds across a single monosaccharide unit (cross-ring cleavages), and the resulting fragments are referred to as A or X ions, and these are useful in linkage and branching determination [81].

In triple quadrupole instruments, the collisions occur in the second quadrupole, and in ion-trap instruments, the ion-trap itself is the location of fragmentation. Since ion-traps can only trap a limited range of masses at the same time, small fragments cannot be trapped simultaneously with their precursor ions. This leads to the “one-third rule,” according to which only fragments with *m*/*z* greater than one third of the precursor *m*/*z* are detected in ion-trap CID-MS/MS [82].

##### Higher-energy collisional dissociation

HCD is a fragmentation technique that is most commonly performed in Orbitrap mass spectrometers. In HCD, precursor ions are isolated in the ion-trap and then transferred to a collision cell (C-trap) where they are fragmented by impact with a collision gas. Fragmentation of the N-glycan during HCD tandem MS, leaving behind a stub consisting of a single GlcNAc, enables unambiguous assignment of N-glycosylation sites of glycoproteins [83]. Since the precursor ions are not measured in the same cell, HCD is not subject to the one third cutoff and can be used to measure small fragment ions, such as the diagnostic oxonium ions. Like CID, HCD is an ergodic process, and the fragmentation patterns are similar to CID (Fig. 6A) [56, 83].

##### Electron transfer dissociation

ETD is a nonergodic fragmentation process and thus differs from CID and HCD in the type of information it provides. ETD preferentially breaks the peptide bonds in glycopeptides, leaving the glycans intact. This makes it useful in determining the sites of glycosylation in a glycopeptide [56]. Figure 6 shows the difference in fragmentation patterns between ETD and HCD, highlighting the power of ETD for site mapping. ETD is accomplished by colliding the positively charged glycopeptide precursor ions with radical anions generated separately from an aromatic reagent compound. Currently, the best reagent compound is fluoranthene [84]. During the collision, an electron is transferred to the precursor ion turning it into a charge-reduced unstable radical cation, which then breaks apart. Since the charge after electron transfer is reduced by one, the precursor ion of the analyte has to be at least doubly charged before the transfer in order to remain in the ion-trap. This can be a significant limitation, especially for small glycopeptides. Fragmentation occurs almost always between C_α_ and N_α_ of each amino acid, producing c and z ions (Fig. 6B). ETD of permethylated glycans generally produces more fragments than CID in the presence of cations like Mg^2+^ [85], especially those resulting from cross-ring cleavages, greatly enhancing the potential for detailed structural analysis.

### Recent advances in glycomics and glycoproteomics

The complexity and microheterogeneity continues to demand technical progress for the structural elucidation of glycoproteins. The focus of most advances is still on MS and on novel sample preparation methods for MS. In recent years, there has been an increased effort to obtain quantitative data along with the traditional qualitative analysis. In this context, methodology developed in proteomics MS is now increasingly applied in glycoprotein characterizations. Considerable advances have been made in sample preparation, fractionation, preconcentration, and quantitation techniques such as the use of label-free and isotopic labeling methods. Instrumental advances include ion mobility-mass spectrometry (IM-MS) and flexible use of fragmentation modes. The increased complexity and enormous data volumes obtained through these modern techniques in glycoprotein characterization necessitated development of better and robust software and computer-aided tools for the data analysis. Simultaneously, the individual steps in glycomics and glycoproteomics are being automated, leading to reduced volumetric and sampling errors, thus improving overall reproducibility and analytical throughput. However, comparison of “normal” versus “aberrant” glycosylation levels of complex biological samples from different sources is still challenging due to inconsistency and low reproducibility in analytical procedures for the characterization of glycoproteins which are often present in very low level in comparison to cellular proteomes [86].

Analytical methodologies using a 96-well plate format to characterize glycoproteins via multistep procedures including protein denaturation, deglycosylation, desialylation, permethylation, and subsequent MALDI-MS profiling were achieved successfully in recent times [87]. Sophisticated glycan derivatization schemes have been incorporated into automated workflow platforms prior to mass spectrometric analysis without affecting repeatability [88]. The traditionally tedious permethylation protocol has been automated recently, and the workflow suitable for high-throughput analysis has been used for the glycan profiling of monoclonal antibodies and recombinant human erythropoietin [89].

Improvements in sample handling of histological tissues and instrument sensitivity along with on-surface multiple enzymatic digestions and microfiltrations, followed by MS, aided sensitive profiling of glycans from tissue samples as small as 1.5 mm in diameter [90]. Research using MS-based imaging enabled the study of spatial and temporal organization of glycans in biological cells and tissues, and this carries the promise of helping in the better understanding of the protein-glycan interactome [86]. Mass spectrometry imaging (MSI)-based research eliminates a lot of the tedious sample handling issues involved in the analysis of isolated samples [91]. Interestingly, these procedures were successfully tuned for the reliable detection of the N-glycan structures on tissues including elucidation of α-2,3 and α-2,6 linkages of their sialic acids [91, 92].

Recently, comprehensive databases of antiglycan reagents including lectins and antibodies are available, and with the increased commercial availability of lectins, including recombinant lectin variants, they have been progressively used for glycoprotein fractionation and glycan-epitope detection [93–95].

The method which is widely used in the pharmaceutical industry for glycan quantification is HILIC HPLC with fluorescence detection of reductively aminated glycans with fluorescent labels, and this procedure is easy to validate under GMP regulations. In one recent report, the hydrophilicity of HILIC materials was improved by using branched copolymer-modified hydrophilic material Sil@Poly(THMA-co-MBAAm) with unique “claw-like” polyhydric groups, and the material showed promisingly improved retention of N-linked glycopeptides [96]. In another recent report, optimized dipeptide-based homo-polymers were employed for glycopeptide enrichment, and the researchers claim that the materials can be used for the separation of glycans with different isomeric glycosidic linkages [97].

The shortcomings of both chemical and enzymatic release of glycans were addressed recently in several innovative studies. Some interesting ones are the development of novel chemical glycan release methods [98–101], optimization of PNGase F release of N-glycans [102], immobilization of PNGase F [103], discovery of broad substrate-specific N-glycosidases [104], and high-throughput methods for glycan release [86].

A new chemical method of N-glycan release and tagging, termed “threshing and trimming” (TaT), was reported recently, where glycoproteins, tissues, or organs were treated with protease pronase (“threshing”) and the generated pool of N-glycopeptides with only one or a few amino acids long was subsequently treated with N-bromosuccinimide (NBS) under mild conditions, leading to oxidative decarboxylation (“trimming”) and release of glycans. This procedure of glycan release is reported to be specific to N-glycans, and the glycans with either nitriles or aldehydes at the reducing end were generated based on reaction conditions. The nitriles are then labeled with 2-AB or the aldehydes with 2-amino-N-(2-aminoethyl)benzamide (AEAB) and are detected with MALDI-MS or HPLC with fluorescence detection [101]. Another interesting method developed by the same group of researchers enabled oxidative release of N-glycans, O-glycans, and glycans from GSLs (glycosphingolipids) by the treatment of samples with household bleach sodium hypochlorite (NaClO). The bleach degrades the proteins while leaving N-, O-, and GSL-associated glycans intact and the liberated glycans are subsequently tagged with different fluorescent tags. This strategy was used to release gram quantities of glycans from various glycoproteins present in egg yolk and porcine tissues. However, some loss of reducing-end N-acetyl glucosamine (GlcNAc) of N-glycans was observed, and the degradation was reported to be dependent on time and temperature of treatment [98]. A process shown to be more efficient than endo-H treatment, in cleaving N-glycans from the peptide while leaving the core GlcNAc amide bond intact, was reported recently which involves treatment of lectin-enriched glycopeptides with a mixture of trifluoromethanesulfonic acid (TFMS) and toluene [99]. A noteworthy recent strategy called “glycoblotting” is a high-throughput technique effecting the release of O-Glycans. The procedure involves treatment of sample with ammonium carbamate at 60 °C for 40 h, followed by washing steps, chemoselective capture on BlotGlyco H hydrazide beads, and subsequent derivatization [105].

Derivatization of glycans with a fluorophore enhances the sensitivity of analysis with both spectroscopic and MS detectors. Moreover, fluorophores increase the hydrophobicity of highly hydrophilic glycans and thereby increases their chromatographic retention in reversed-phase liquid chromatography. In yet another novel strategy, monosaccharides were labeled with the fluorescent tag, 2-pyridylfuran (2-PF), introduced by reaction with 1,3-di(2-pyridyl)-1,3-propanedione (DPPD). HPLC analysis of these derivatives achieved sub-femto mole levels of detection [106]. Another recent study reported a new improved label, RapiFluorMS (RFMS), which enables rapid labeling of N-glycans, released as glycosylamine, at their reducing end immediately after PNGase F treatment. The label bears dual functional centers: a quinone moiety as fluorophore and a tertiary amine for strong positive-mode ionization [107]. The common problem of fucose migration in LC-MS tandem MS fragmentation of native or reductively labeled glycans from the antenna to the core or vice versa was recently addressed by reducing-end labeling with procainamide hydrochloride. Moreover, during LC-MS analysis, it improved ionization of labeled glycans 10–50 times in comparison to 2-AB labeling [108]. The chemical release of O-glycans via β-elimination results in a reduced form of glycan which are not easy to be derivatized. This problem was addressed in a study where a one-pot simultaneous release and labeling of O-glycans was achieved by performing β-elimination in the presence of 1-phenyl-3-methyl-5-pyrazolone (PMP), leading to PMP-labeled O-glycans [109]. A technique for the selective enrichment of O-GlcNAc-modified proteins was developed by Griffin et al., in which an azide-bearing monosaccharide was chemoenzymatically attached to O-GlcNAc modifications of proteins and the azide functional groups of introduced azide-bearing monosaccharides were subsequently probed by a linker via copper(I)-catalyzed azide-alkyne cycloaddition (CuAAC) [110].

Quantitative techniques are increasingly applied in the field of glycomics and glycoproteomics, and numerous researchers in academia and industry have currently shifted their attention to MS-based relative and absolute quantification of glycoconjugates. Chemical labeling with an isotope tag is one of the most popular method for MS-based relative quantitation because the isotope tag does not affect chromatographic separation or ionization in MS while providing an isotopic mass shift to differentiate the labeled molecules. The “light” and “heavy” isotope-labeled glycans, where isotopic tags with lower mass and higher mass are used, respectively, are mixed at 1:1 ratio and the corresponding MS peak height is monitored for the estimation of relative quantity. Commonly, the isotope tags are incorporated into glycans chemically during either permethylation of N- and O-linked glycans (using reagents such as CD_3_I or ^13^CH_3_I vs ^12^CH_3_I) or reductive amination of N-glycans [111]. Incorporation of ^18^O isotope on N-glycans and at the site of N-glycans attachment to glycopeptide via enzymatic de-glycosylation with PNGase F in H_2_^18^O is also a common method of isotopic labeling [112]. Metabolic labeling of glycans with sialic acid and GalNAc analogues bearing isotopic atoms and the relative quantitation of glycans which are tagged by these isotopes via LC-MS/MS analysis (IsoTag) was another recent endeavor for the glycopeptide enrichment and isotope tagging [113]. Introduction of multiplex aminoxyTMT reagents enabled efficient relative quantitation of carbohydrates by improving glycan ionization efficiency and analytical throughput [114]. Similarly, tags termed Quaternary Amine Containing Isobaric Tag for Glycan (QUANTITY) which can completely label glycans and generate strong reporter ions were also reported for the labeling of up to four samples at a time (4-plex) and simultaneous analysis for the relative quantification of glycans [115]. Multiple reaction monitoring (MRM) and label-free quantitation, which does not involve laborious derivatization procedures, are the emerging novel ways for the relative quantitation of glycans and glycoforms of glycoproteins [116].

The most common modes of ionization in glycan and glycopeptide characterization currently are MALDI and ESI. However, each of them has their own disadvantages. One of the most common disadvantage of ESI is in-source fragmentation, which can lead to misinterpretation and poor sensitivity. In order to address this problem, a new technique of using subambient pressure ionization with a nanoelectrospray (SPIN) source was developed. Here, the ESI emitter which was kept at atmospheric pressure was moved to the first vacuum stage of the mass spectrometer and placed at the entrance of the electrodynamic ion funnel to enhance the collection of the entire electrospray plume [117]. Since this technique improves the MS sensitivity and makes the ionization conditions gentler, glycan coverage was reported to be increased by 25% relative to conventional ionization techniques. This modification enabled the detection of heavily sialylated and polysialylated glycans from human serum [86, 118].

Another robust technique developed recently in MS-based fragmentation is electronic excitation dissociation (EED) that occurs at an electron energy of >9 eV and provides rich structural information through MS, in comparison to CID-based fragmentation of permethylated and reducing end-labeled glycans [119]. The collision energy required for the optimal fragmentation of glycan and peptide portion of a glycopeptide differs considerably, and one recent technology termed collision energy stepping CID allows simultaneous acquisition of MS/MS spectra of glycopeptide at lower and higher collision energies [120].

Despite using multiple tandem fragmentation techniques, glycopeptide MS data interpretation is mostly ambiguous, and thus, in order to lower false discovery rates (FDR), data-dependent decision trees of sequential fragmentation steps of glycopeptides such as HCD-product dependent-ETD/CID workflow utilizing tribrid Orbitrap mass spectrometers are currently employed. The data interpretation was facilitated by such improved tandem MS data and development of novel algorithms that employs machine-learning to predict N-glycopeptides [86, 121].

Other notable recent progresses in MS-based instrumentations for glycoprotein characterization are as follows: development of spectral libraries of sodiated oligosaccharides by sequential MS fragmentation in the positive-ion mode [122]; introduction of novel fragmentation method such as ultraviolet photodissociation (UVPD) for the improved spectral data of glycans [123]; employing negative-ion mode in glycan fragmentation in order to obtain more diagnostic cross-ring fragments [124]; development of promising applications of IM-MS in the discrimination of linkage and position isomers, identification of glycosylation sites, and information on potential conformational changes that are induced from protein-glycan interactions; introduction of CID prior to ion mobility separation for the discrimination of epimeric oxonium ions from D-GalNAc and D-GlcNAc glycoforms; discrimination of sialic acid linkage isomers (α-2-3 and α-2-6 linked sialic acid) by the use of traveling-wave ion mobility spectrometry (TW-IM-MS) [125]; and finally, increased use of capillary zone electrophoresis for the unprecedented separation efficiencies, resolution, and sensitivities in the characterization of glycoconjugates from charged biomolecules [88].

In order to promote critical evaluation of experimental protocols, dissemination of data sets for reproducibility, and comparison of results obtained in different laboratories, an initiative termed minimum information required for a glycomic experiment (MIRAGE), was established in 2011. MIRAGE provides guidelines for data reporting of mass spectrometry, liquid chromatography, sample preparation, and data handling [126–128].

### Resources

#### General resources

The Complex Carbohydrate Research Center at the University Georgia has comprehensive analytical service, collaboration, and hands-on training resources in the area of glycoproteins, polysaccharides, and proteoglycans derived from animal, plant, and microbial origin, as well as databases of PMAA GC-MS spectra for linkage analysis [129], and a modular software tool, GRITS Toolbox [130], for help in processing, annotating, and archiving of glycomics data (see “Mass spectrometry software and tools” section). The Consortium for Functional Glycomics (CFG) is a comprehensive resource for functional glycomics research that contains data from experiments that have been performed by the CFG, including glycan array screening, glycan profiling, glycogene microarray screening and phenotyping of glycogene mouse strains, and detailed information about glycan structures, glycan-binding proteins, and glycosyltransferases [131]. *Essentials of Glycobiology*, a comprehensive textbook in glycobiology, is a useful book published by the Consortium of Glycobiology in collaboration with the National Library of Medicine/National Center for Biotechnology Information (NLM/NCBI) and Cold Spring Harbor Laboratory (CSHL) Press [6]. This book is freely accessible online [132] and is an invaluable resource for understanding the background and context of glycoproteins. A third and significantly updated edition of this book is scheduled to be released later this year.

#### Common representation of glycans

**Fig. 10:**
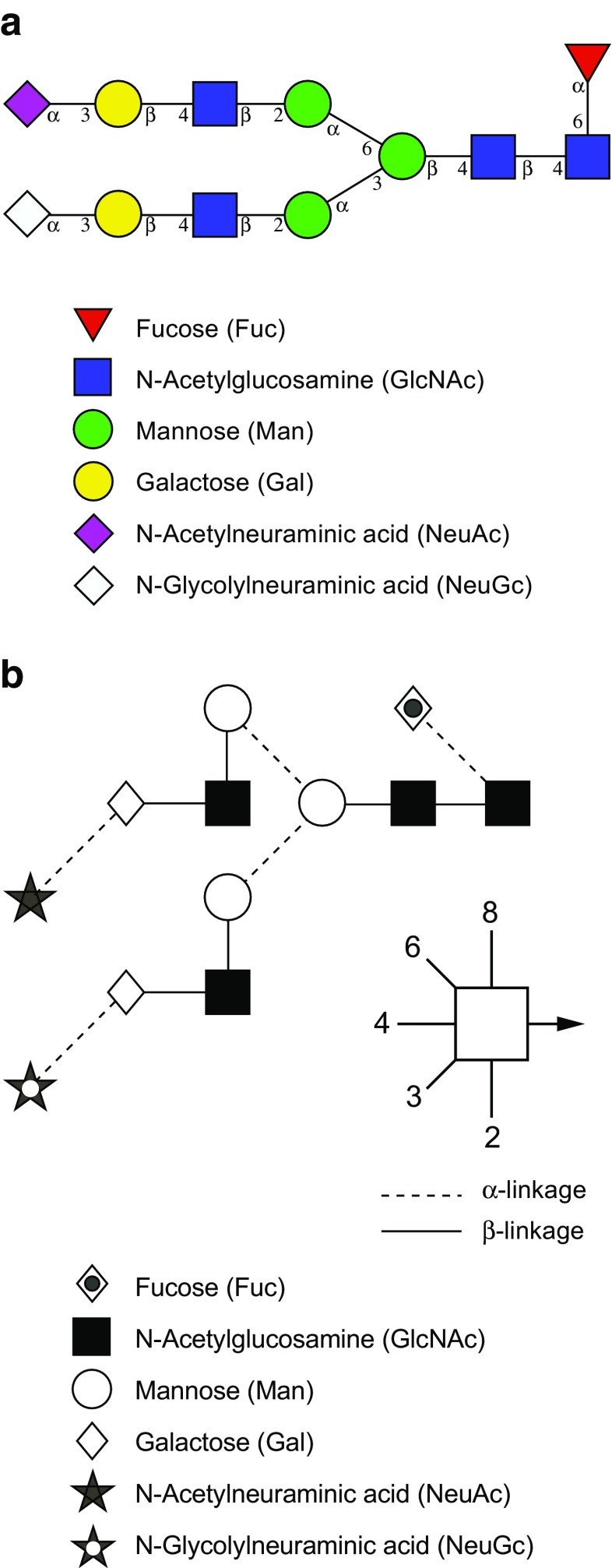
Common representation of glycans. **A** CFG notation [46]. **B** Oxford-Dublin notation [47]

In order to represent the carbohydrate structures in a simple and accurate way, different graphical representations or “cartoons” for glycan structures were proposed. Two common notation schemes are CFG nomenclature scheme [46] and the Oxford-Dublin system (Fig. 10) [47]. In CFG notation, different geometric symbols are used to represent different monosaccharides, and different monosaccharides that have same mass are assigned the same shape. For example, hexoses are represented by circles and the isomers of hexoses are indicated by different colors, such as blue for glucose, green for mannose, and yellow for galactose. Likewise, derivatives of a given monosaccharide are assigned the same color but different shapes; for example, glucose as blue circle and GlcNAc as blue square. Since some confusion may arise when using black and white images of these CFG notations, the Oxford-Dublin system uses different shapes for each monosaccharide (Table 2).

**Table 2:** A glossary of glycoscience terms used in this tutorial

Monosaccharide nomenclature	Glc	Glucose
	GlcNAc	2-*N*-acetylglucosamine
	Gal	Galactose
	GalNAc	2-*N*-acetylgalactosamine
	Man	Mannose
	Fuc	Fucose
	NeuAc	5-*N*-acetylneuraminic acid
	Sialic acid	A general term for neuraminic acids
	Hex	Hexose (e.g., Glc or Gal or Man)
	HexNAc	*N*-acetylhexosamine (e.g., GlcNAc or GalNAc)
	dHex	Deoxyhexose (e.g., Fuc)
Other terminologies	Reducing end	The reducing end of a glycan is the monosaccharide with a free anomeric carbon that is not involved in a glycosidic bond and is thus capable of converting to the open ring form
	Permethylation	Derivatization of all OH to O-methyl and NH to N-methyl
	Glycosidic bond	Covalent bonds associated with carbohydrates
	Cross-ring cleavage	Fragmentation across a monosaccharide ring
	Scar	A nonmethylated hydroxyl group of glycan exposed by tandem MS fragmentation of a glycosidic bond in a permethylated glycan
	β-Elimination	Release of *O*-glycosidic linkages between glycans and the β-hydroxyl groups of serine or threonine

#### Mass spectrometry software and tools

Several tools are available that assist in predicting/interpreting glycan structure from MALDI or ESI-MS^*n*^ data or glycopeptide structure from LC-MS/MS data. The following open source tools are commonly used for characterization of glycosylation by mass spectrometry.

GlycoWorkbench [133] is a tool used for the drawing of glycan structures and automatically matching these models and their theoretical fragments with the experimental mass spectra. This tool provides complete support to the routine interpretation of glycomic mass spectrometric data and comprises several features to annotate MS data. The visual editor module of GlycoWorkbench, the GlycanBuilder, supports rapid assembly of graphical representations of glycan structures. GlycanBuilder enables sequential addition of monosaccharides starting from the reducing end, addition of modifications or reducing-end markers, and simultaneously computing of corresponding theoretical *m*/*z* value of glycan structures. Chemical derivatizations of the glycan such as permethylation, sulfation, phosphorylation, and acetylation can also be incorporated into glycan structures and calculate their theoretical *m*/*z*. GlycoWorkbench can also compute various possible monosaccharide compositions, with or without modifications, for the unknown theoretical *m*/*z* values with a defined set of parameters, match them with the experimental *m*/*z* value from a database and generate report in which compositions are listed together with the *m*/*z* accuracy. The software is available publicly for download from the EUROCarbDB web site [26].

GRITS Toolbox [130] is a modular software suite that helps in processing, annotating, and archiving of glycomics data, and thereby assists in determining structures of glycans from the MS and MS/MS data. GRITS Toolbox can process different types of open file format MS data of released glycans with different chemical derivatives such as permethylation and various reducing terminal modifications and annotate them using the integration annotation module Glycomics Elucidation and Annotation Tool (GELATO). It proposes glycan structures as annotations by using a set of databases which have been curated using Qrator software from a combination of external literature and database references, user annotations, and canonical trees [25].

Online web tools such as GlycoMod are also useful in determining all possible compositions of both free glycans and glycans attached to the peptides, based on their experimentally determined masses [28]. The program also predicts the composition of oligosaccharides derived from any glycopeptide comprised of either underivatized, methylated, or acetylated monosaccharides, or with a derivatized reducing terminus. The composition of a glycan on a glycopeptide can also be computed if the sequence or mass of the peptide is known. The program matches the experimentally determined masses against all the predicted protease-produced peptides of known protein amino acid sequences or proteins on Uniprot databases, which have the potential to be glycosylated with either N- or O-linked glycans, and generate glycan compositions. GlycanMass [134] is another web-based program which calculates the mass of a glycan structure from its oligosaccharide composition.

Commercial software such as Byonic and SimGlycan are powerful tools for the rapid detailed analysis of complex N- and O-linked glycan structures from the LC-MS/MS data of glycopeptides. Byonic enables search for tens or even hundreds of variable modification types on peptides simultaneously, including unanticipated or even unknown modifications. Glycopeptide search using Byonic allows the identification of glycopeptides without prior knowledge of glycan masses or glycosylation sites [24]. SimGlycan predicts the structure of glycans and glycopeptides using experimental mass spectrometric MS, MS/MS, and Multi Stage/Sequential mass spectrometry data (MS^*n*^, *n* > 2); matches them with its own database of theoretical fragments; and generates a list of probable candidate structures. It can also analyze mass spectrometry data for released glycans that are underivatized, permethylated, and reducing-end modified, and assists in resolving heterogeneity, branching patterns, and isobaric oligosaccharide structures through MS^*n*^ data analysis. Carbohydrate residues that are modified with substituents such as sulfate, phosphate, ethanolamine, etc. can also be identified from complex glycan structures including glycosaminoglycans [27].

Tools such as NetNGlyc [135] and NetOGlyc [136] are valuable web servers that work on trained artificial neural networks for the prediction of sites of N-linked and O-linked glycosylation, respectively, on glycoproteins.

## Concluding remarks

The field of analytical glycobiology has evolved substantially during the past decade. Recent discoveries about key roles that protein glycosylation plays in cellular physiology and disease processes have drawn the attention of more researchers toward the identification and characterization of the glycome associated with proteins. Scientific endeavors, such as (i) the pursuit of novel disease biomarkers, (ii) recombinant glycoprotein therapeutics, (iii) cell signaling and immunology, and (v) microbial and plant biology, necessitated the development of high-throughput and in-depth analysis methodologies for the characterization of protein glycosylation.

This tutorial has summarized the most common and reliable, yet challenging, analytical techniques prevalent in the fields of glycomics and glycoproteomics. Emphasis has been placed on the enrichment and fractionation of highly heterogeneous glycoprotein samples, enzymatic and chemical treatments, high sensitive detection via derivatizations, detailed analysis through technologically advanced instrumental methods, and finally, the interpretation of glycan structure on glycoproteins through valuable resources and bioinformatics tools.

Implementation of advanced analytical methodologies that can help in the hyphenation of complementary approaches like glycomics and glycoproteomics would pave the way for future explorations of physiological interactions associated with glycomes and glycoproteomes.
